# The BLOC-1 Subunit Pallidin Facilitates Activity-Dependent Synaptic Vesicle Recycling

**DOI:** 10.1523/ENEURO.0335-16.2017

**Published:** 2017-02-08

**Authors:** Xun Chen, Wenpei Ma, Shixing Zhang, Jeremy Paluch, Wanlin Guo, Dion K. Dickman

**Affiliations:** 1 Department of Biological Sciences, University of Southern California, Los Angeles, CA 90089; 2 Neuroscience Graduate Program, University of Southern California, Los Angeles, CA 90089

**Keywords:** BLOC-1, Drosophila, endocytosis, endosome, neuromuscular junction, synaptic vesicle

## Abstract

Membrane trafficking pathways must be exquisitely coordinated at synaptic terminals to maintain functionality, particularly during conditions of high activity. We have generated null mutations in the *Drosophila* homolog of pallidin, a central subunit of the biogenesis of lysosome-related organelles complex-1 (BLOC-1), to determine its role in synaptic development and physiology. We find that Pallidin localizes to presynaptic microtubules and cytoskeletal structures, and that the stability of Pallidin protein is highly dependent on the BLOC-1 components Dysbindin and Blos1. We demonstrate that the rapidly recycling vesicle pool is not sustained during high synaptic activity in pallidin mutants, leading to accelerated rundown and slowed recovery. Following intense activity, we observe a loss of early endosomes and a concomitant increase in tubular endosomal structures in synapses without Pallidin. Together, our data reveal that Pallidin subserves a key role in promoting efficient synaptic vesicle recycling and re-formation through early endosomes during sustained activity.

## Significance Statement

The speed and efficiency of synaptic vesicle recycling and re-formation are critical to maintain a functional synaptic vesicle pool during intense neuronal activity, yet the mechanisms that achieve this remain enigmatic. We show that the biogenesis of lysosome-related organelles complex-1 (BLOC-1) component Pallidin promotes rapid and efficient synaptic vesicle recycling through endosomal intermediates. Further, Pallidin is necessary for the integrity of Rab5-positive synaptic endosomes during sustained neurotransmission.

## Introduction

Synaptic vesicle trafficking must be tightly coordinated to ensure rapid, efficient, and reliable replenishment following exocytosis, particularly during intense levels of neuronal activity. To accomplish this, synapses are endowed with a variety of mechanisms to ensure fast and robust synaptic vesicle recycling. It is clear that there are slow and fast forms of synaptic vesicle endocytosis (Sudhof, 2004; Smith et al., 2008; Kononenko and Haucke, 2015), and that dynamin is necessary for vesicular fission (Ferguson and De Camilli, 2012; Rizzoli, 2014). There are at least three fundamental trafficking pathways for synaptic vesicles at the synapse. These include clathrin-mediated endocytosis and direct re-formation of synaptic vesicles to the fusion competent pool (Murthy and De Camilli, 2003; McMahon and Boucrot, 2011), trafficking of synaptic vesicles through endosomal intermediates (de Hoop et al., 1994; Salem et al., 1998; Wucherpfennig et al., 2003; Voglmaier et al., 2006; Brown et al., 2009; Hoopmann et al., 2010; Uytterhoeven et al., 2011; Kononenko and Haucke, 2015), and bulk endocytosis pathways (Murthy and De Camilli, 2003; Clayton and Cousin, 2009a; Wu et al., 2014; Soykan et al., 2016). Further, following re-formation of functional synaptic vesicles through these routes, these vesicles must be organized into pools of varying locations, releasable states, and biochemical interactions that ultimately maintain neurotransmission and determine synaptic strength (Rizzoli, 2014). Although several of the key molecules involved in the essential steps of membrane trafficking have been identified, much less is known about how synaptic vesicle recycling is dynamically modulated to meet the changing demands of synapses during sustained neurotransmission. More specifically, how the demands of synapses during intense stimulation are transduced to enable adaptive modulations in the speed and destinations of synaptic vesicle transport remains enigmatic.

Endosomes are key nodes of signaling and trafficking during synaptic vesicle recycling. Although Rab5 and other factors are known be involved in synaptic endosomal function (Wucherpfennig et al., 2003; Uytterhoeven et al., 2011; Kononenko and Haucke, 2015), our understanding of synaptic vesicle trafficking through endosomes remains incomplete. The biogenesis of the lysosome-related organelles complex-1 (BLOC-1) complex has been implicated in endosomal sorting in a variety of tissues, but its role at synapses is not established. The BLOC-1 is composed of eight subunits, Blos1, Blos2, Blos3, Snapin, Dysbindin, Pallidin, Muted, and Cappuccino (Falcón-Pérez et al., 2002; Ciciotte et al., 2003; Li et al., 2003; Starcevic and Dell'Angelica, 2004; Lee et al., 2012). Mutations in human *pallidin* and other BLOC-1 components are associated with Hermansky–Pudlak syndrome, a disease in which trafficking and biogenesis of platelets, lysosomes, and other endosomal organelles are impaired (Wei, 2006). In addition, mutations in the BLOC-1 components *dysbindin*, *muted*, and *blos3* have been associated with schizophrenia (Straub et al., 2002; Morris et al., 2008; Ryder and Faundez, 2009; Ghiani and Dell'Angelica, 2011). The central component of this complex is Pallidin, which biochemically interacts with Dysbindin, Blos1, and Cappuccino (Starcevic and Dell'Angelica, 2004; Lee et al., 2012). However, the role of Pallidin and BLOC-1 in the nervous system in general, and at synapses in particular, is not understood.

The fruit fly *Drosophila melanogaster* is an attractive model system to elucidate the role of *pallidin* at synapses. The fly genome encodes single orthologs of each vertebrate BLOC-1 subunit, including *pallidin* (Cheli et al., 2010; Mullin et al., 2015), and there is evidence for similar interactions between subunits (Cheli et al., 2010). *Drosophila blos1* has been implicated in pigmentation trafficking in photoreceptors (Cheli et al., 2010), while *dysbindin* and *snapin* were found to be necessary for presynaptic homeostatic plasticity (Dickman and Davis, 2009; Dickman et al., 2012), an adaptive form of synaptic plasticity that leads to an increase in presynaptic release in response to perturbation of postsynaptic neurotransmitter receptors, maintaining stable levels of synaptic strength (Frank, 2014; Davis and Müller, 2015). Finally, in addition to the sophisticated genetic approaches available in *Drosophila*, the fly neuromuscular junction (NMJ) permits powerful electrophysiological, imaging, and cell biological tools with the potential to reveal the functions of *pallidin* and other BLOC-1 subunits in synaptic structure and function.

To gain insight into the role of BLOC-1 at synapses, we have generated mutations in the *Drosophila* homolog of *pallidin*. Our characterization of synaptic development and physiology in these mutants has revealed that Pallidin is localized to cytoskeletal structures at synaptic terminals but is surprisingly dispensable for synaptic growth, structure, baseline function, and homeostatic plasticity. However, *pallidin* is necessary to replenish depleted synaptic vesicles during high levels of activity by promoting the rapid trafficking and re-formation of vesicles through endosomal intermediates. Thus, Pallidin has an important function in maintaining and replenishing the activity-dependent synaptic vesicle pool.

## Materials and Methods

### *Drosophila* genetics and molecular biology

*Drosophila* stocks were raised at 25°C on standard molasses media containing 12-g inactive yeast, 60-g cornmeal, 6-g agar, 74-ml molasses, 5.5-ml propionic acid, and 11-ml tegosept (10% w/v) per liter. It was communicated to us that raising *pldn* mutants in an alternative food source, containing 36-g inactive yeast, 89-g cornmeal, 6.6-g agar, 89-ml molasses, 6.6-ml propionic acid, and 17.8-ml tegosept (10% w/v) per liter results in changes in synaptic growth in *pldn* and other BLOC-1 mutants that were not observed using the recipe used in this study (V. Faundez, personal communication). The reasons for this distinction are under active investigation by Dr. Faundez.

The precise deletion of the *pallidin* locus was generated using flippase (FLP) recombinase-mediated recombination between pairs of transposon-based FRT sites, as described in the DrosDel Collection (Parks et al., 2004). Specifically, two transposons flanking the *pallidin* locus, *pBac^f05716^* and *pBac^f05753^* (Thibault et al., 2004), were obtained from the Bloomington *Drosophila* Stock Center. Each contained flippase recombination target (FRT) sites in the correct orientation to permit a precise deletion. Following FLP-mediated recombination and excision of the remaining hybrid transposon, we confirmed the deletion by PCR using the following primers: forward primer 5’-GTCATTGGGTGCAAAGTGCTC; reverse primer 5’-CTCCCGAGCTGCATGTTGAATC. This revealed that bases 11,689,565 to 11,691,527 on chromosome 3L were deleted. We named this mutant allele *pldn^Δ1^*. The transcript of the gene located 5’ to pallidin is not perturbed, while an estimated 591 bases of the 3’UTR of *sugb*, the gene located 3’ to the *pldn* locus, is deleted in *pldn^Δ1^*. This region overlaps with the predicted pallidin 3’UTR, making it difficult to conclude whether the neighboring gene is impacted. The *w^1118^* strain was used as the wild-type control unless otherwise noted, as this was the genetic background in which all genotypes were bred. *UAS-GFP-myc-2xFYVE* was obtained from Marcos Gonzalez-Gaitan (Wucherpfennig et al., 2003), and *blos1^ex2^* (Cheli et al., 2010) was a gift from Esteban Dell’Angelica (UCLA). *Df(3L)BSC675* (*pldn* deficiency) and *dysbindin^e01028^* (refered to as *dysb^1^*) was obtained from Bloomington *Drosophila* Stock Center (Bloomington, IN), as were all others unless otherwise noted. Standard second and third chromosome balancers and genetic strategies were used for all crosses and for maintaining mutant lines. For all experiments, animals of either sex were used unless otherwise specified.

We obtained cDNA of the entire *pallidin* open reading frame from the Berkeley *Drosophila* Genome Project (IP05492). We cloned the *pallidin* cDNA into the pACU2 vector (Han et al., 2011) using standard cloning methods (5’ EcoR1 restriction enzyme; 3’ Xba1 restriction enzyme) to generate *UAS-pallidin*. For generation of *UAS-pallidin-3xflag* we inserted synthesized 3xflag coding sequence: GCATGGATTACAAGGATCACGACGGCGATTACAAGGATCACGACATCGATTACAAGGATGACGATGATAAGTAA and cloned the sequence into the pACU2 vector using standard cloning methods (5’ Nde1 restriction enzyme; 3’ Spe1 restriction enzyme), we then cloned the *pallidin* cDNA into this pACU2 vector that contains the 3xflag sequence using standard cloning methods (5’ EcoR1 restriction enzyme; 3’ Xba1 restriction enzyme). These constructs were sequenced and sent to BestGene for recombination-mediated insertion into the VK18 (Venken et al., 2006) recombination site on the second chromosome.

### Immunochemistry and immunoblot analysis

Wandering third-instar larvae were dissected with pins on a Sylgard dish, fixed in Bouin’s fixative (Sigma, HT10132-1L) or 4% paraformaldehyde in PBS (Sigma, F8775), and immunostained with primary antibodies diluted in PBST (PBS supplemented with 0.05% Triton X-100, Sigma, X100) at 4°C overnight or room temperature for 30 min, followed by 3 times washing with PBST and secondary antibody incubation at room temperature for 2 h, samples were then washed three times with PBST and mounted on a glass slide for imaging. To generate the Pallidin antibody used in this study, we synthesized a peptide consisting of amino acids GRQNKTYIDLSKEKYK of the Pallidin amino acid sequence (amino acids 80-95). This peptide was conjugated to KLH and injected into rabbits to obtain immunosera that was subsequenctly affinity purified (Yenzym). The following primary antibodies were used at the indicated dilutions: mouse anti-BRP nc82 1:100 (Developmental Studies Hybridoma Bank; DSHB, RRID:AB_2314867), rabbit anti-DLG 1:10,000 (Koh et al., 1999), mouse anti-SYN 1:20 (3C11; DSHB, RRID:AB_2313867), guinea pig anti-vGlut (1:200), goat anti-HRP 1:200 (directly conjugated to Alexa Fluor 647; Jackson ImmunoResearch), mouse anti-Futsch 22C10 1:50 (Developmental Studies Hybridoma Bank; DSHB, RRID:AB_528403), and rabbit anti-PLDN 1:200. Alexa Fluor 488- and Cy3-conjugated donkey secondary antibodies (Jackson ImmunoResearch) were used at 1:400. Images were acquired with a Nikon A1R Resonant Scanning Confocal microscope equipped with NIS Elements software and a 100× APO 1.4NA oil immersion objective. Settings were optimized for detection without saturation of the signal. Z-stacks were obtained using identical settings within each experiment and maximal intensity projection of each Z-stack were used for analysis. Bouton numbers were quantified directly from preparations stained for Synapsin under a confocal microscope on muscle 6 and 7 of segment A3. BRP number and density and Pldn intensity were quantified using NIKON NIS-Elements Advanced Research software, intensity values were measured as mean intensity. BRP number, density, and HRP area were quantified from images of muscle 4 segment A3. Pldn intensity was quantified from images of muscle 6 and 7 of segment A2.

For GFP-2XFYVE live imaging, animals were dissected in 0.4 mM Ca^2+^ HL-3 and incubated in modified HL-3 with 2 mM Ca^2+^, 90mM K^+^(high K^+^) HL-3 for 5 min for high K^+^ stimulation. GFP-2XFYVE and Cy3-HRP (Jackson ImmunoResearch, 1:400) were imaged using a Zeiss LSM700 confocal microscope equipped with Zen software using a 63× 1.0 NA water immersion objective. Analysis of GFP-2XFYVE puncta, including the density (number of GFP-2XFYVE puncta/HRP area), size, and intensity, was performed using ImageJ (NIH). For analysis of size and intensity, the number of puncta after high K^+^ was normalized to each genotype’s puncta number at rest.

For immunoblot analysis, 50 adult heads were collected and homogenized in 100-μl lysis buffer (10 mM HEPES, pH 7.4; 150 mM NaCl; protease inhibitors (Roche); and 1% Triton X-100). A total of 10 μl of protein lysate was separated by SDS-PAGE and transferred to PVDF membranes. Western blot analysis was performed according to manufacturer’s protocols. SuperSignal West Femto Maximum sensitivity substrate (Thermo Scientific) were used for x-ray film-based band visualization. The film were scanned and band intensities were quantified with ImageJ (NIH). The following antibodies were used: rabbit anti-PLDN 1:1000, mouse anti-α-tubulin 1:2000 (T6199, Sigma-Aldrich).

## Electrophysiology

Electrophysiology was performed using a Zeiss Axioscope AX10 fixed stage microscope equipped with a 40× 0.8 NA water-dipping objective. Third-instar larvae were dissected and bathed in a modified HL-3 saline: 70 mM NaCl; 5 mM KCl; 10 mM MgCl_2_; 10 mM NaHCO_3_; 115 mM sucrose; 5 mM trehelose; 5 mM HEPES, pH 7.2; and a calcium concentration of 0.4 mM unless otherwise specified. Sharp electrode current-clamp recordings were performed on muscles 6 and 7 in abdominal segments A2 or A3. Severed ventral nerves were stimulated using a 5V command pulse at 3 ms stimulus duration through pClamp software to an Isoflex stimulation unit (A.M.P.I.). Data were acquired using an Axoclamp 900A amplifier, digitized using a Digidata 1440A, and controlled using pClamp 10.5 software (Molecular Devices). Electrophysiological sweeps were sampled at a rate of 10 kHz and filtered at 400 Hz. Data were analyzed using MiniAnalysis (Synaptosoft), SigmaPlot (Systat Software), GraphPad Prism, Microsoft Excel, and SPSS 13.0. Quantal content was calculated for each individual recording by calculating the average EPSP, average miniature EPSP (mEPSP), and corrected for nonlinear summation for the calcium-cooperativity analysis, using equation QC_corrected_ = (EPSP/mEPSP)(1-EPSP/V_0_)^−1^, where V_0_ = (reversal potential – resting potential) (Martin, 1955). Corrected and noncorrected quantal content values were compared in all experiments, and in no case did quantal content values change any conclusion. A recording electrode (15- to 30-MΩ resistance) filled with 3 M KCl was used, and data were only analyzed from cells with a resting potential more hyperpolarized than -60 mV, input resistance of at least 5 MΩ, and resting potentials that did not deviate by more than 5% for the duration of the recording. For acute pharmacological homeostatic challenge, semi-intact preparations, with the CNS, and gut left intact, were perfused with Philanthatoxin-433 (20 µM in HL3, Sigma) for 10 min followed by full dissection and electrophysiological recording as described previously (Frank et al., 2006). For failure analysis, recordings were performed in 0.1 mM Ca^2+^ saline, and percent failure of EPSP was calculated from 40 stimulation trials in each recording. To determine asynchronous release rates, mEPSP frequency was calculated for the 2-s period immediately following EPSP stimulation of 30 EPSP trials during each recording.

Two electrode voltage clamp recordings were used to determine the readily releasable pool (RRP) size. Recordings were made from cells with input resistances ≥5 MΩ and membrane potentials between −55 and −70 mV in modified HL-3 saline containing 3 mM extracellular calcium. Intracellular electrodes with resistances of 10–30 MΩ filled with 3 M KCl were used. The holding potential was −70 mV. EPSC amplitudes during a stimulus train (60 Hz, 60 stimuli) were calculated as the difference between the peak and baseline before stimulus onset of a given EPSC. The number of release-ready vesicles was obtained by back-extrapolating a line fit to the linear phase of the cumulative EPSC plot (the last 30 stimuli) to time 0 and dividing the cumulative EPSC amplitude at time 0 by the mean mEPSC amplitude recorded in the same cell, as described previously (Muller et al., 2012).

### Electron microscopy

EM analysis was performed as described previously (Kaufmann et al., 2002). Wandering third-instar larvae were dissected in Ca^2+^-free HL-3 saline (rest) or 2 mM Ca^2+^, 90 mM K^+^(high K^+^) modified HL-3 saline, then fixed in 2.5% paraformaldehyde/5.0% glutaraldehyde/0.06% picric acid/0.1 M cacodylate buffer for ∼18 h at room temperature. Fillets were rinsed three times for twenty minutes in 0.1 M cacodylate buffer. The larval pelts were then placed in 1% osmium tetroxide/potassium ferrocyanide mix buffer (1% OsO_4_,1.5% K_4_[Fe(CN)_6_] in water) for 1 h at room temperature. After rinsing and dehydration in an ethanol series, samples were cleared in propylene oxide and infiltration with half propylene oxide and half TAAB resin overnight at 4°C . The following day, samples were embedded in fresh TAAB resin. EM sections were obtained on a JEOL 1200EX microscope at the EM Facility of Harvard Medical School. The 6/7 muscle region was located by taking 0.5-μm sections and the bouton regions were located by taking 90-nm sections until boutons were identified. The blocks were then trimmed and serial sectioned at a 60-nm thickness for approximately 240 sections. The sections were mounted on Formvar-coated single slot grids and viewed at a 25,000× magnification. Measurements were taken to scale with 10× lupe/mm. Images were analyzed blind to genotype using ImageJ (NIH) and Adobe Photoshop (Adobe Systems) software. The 3D reconstruction model was generated using IMOD software (Kremer et al., 1996).

### Statistical analyses

Statistical analyses were performed using SPSS 13.0 software (IBM). Student’s *t* test was used to compare two groups. The one-way ANOVA plus *post hoc* LSD (with equal variances) or Tamhane’s T2 (with unequal variances) tests were used to compare three or more groups. Statistical significance was defined as *p* < 0.05 levels. All data are presented as group means ± SEM.

## Results

### Generation and analysis of mutations in the *Drosophila* homolog of *pallidin*

**Figure 1.**
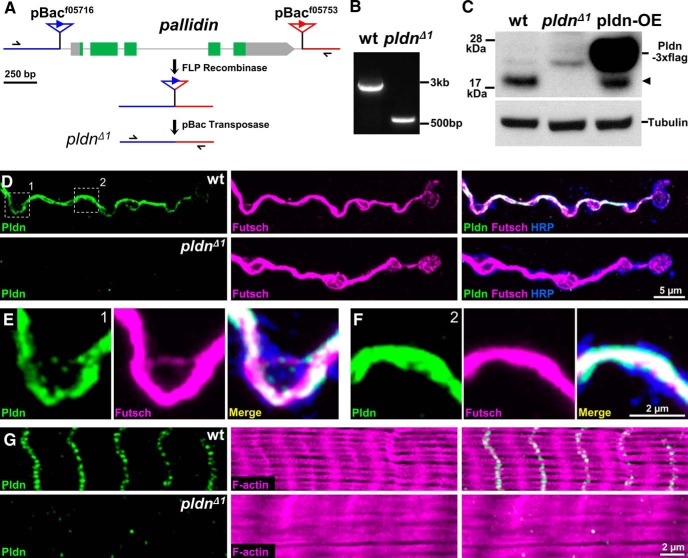
Generation of *pallidin* null mutations and synaptic localization of Pldn protein. ***A***, Schematic of the workflow utilized to generate the excision of the *pallidin* locus. Black arrows indicate primers used for PCR confirmation of the excision. ***B***, PCR confirmation of the deletion of the *pldn* locus. ***C***, Immunoblot analysis of adult heads lysates from wild type (*w^1118^*), *pldn^Δ1^* mutants (*w^1118^*;*pldn^Δ1^*), and neuronal *pldn* overexpression (pldn-OE; *c155-Gal4*/*Y*;*UAS-pldn-3xflag*/*+*), which reveals a band running at 19 kDa, the predicted molecular weight of *Drosophila* Pldn. This band (indicated by arrowhead) is absent in *pldn^Δ1^* and increased in pldn-OE. Anti-α-tubulin immunoblot was used as loading control. ***D***, Representative images of third-instar larval NMJs from wild-type and *pldn^Δ1^* mutants immunostained for Pldn (green) and the neuronal microtubule marker Futsch (magenta). The neuronal membrane is immunolabeled with anti-HRP (blue). ***E***, Magnified images of area 1 and area 2 (***F***) marked in ***D***, exhibiting a high degree of colocalization between Pldn and Futsch. ***G***, Representative images of third-instar larval muscle immunostained for Pldn (green) and F-actin (phalloidin; magenta), showing Pldn localization to the muscle Z band.

Pallidin is a core component of the BLOC-1 complex (Li et al., 2003; Starcevic and Dell'Angelica, 2004; Lee et al., 2012) but has not been studied in *Drosophila*. We identified independent transposon insertions flanking the *Drosophila pallidin* locus (*CG14133*; hereafter abbreviated *pldn*). These *piggyBac* transposons fortuitously carried FRT sequences in the same orientation, enabling FLP-mediated recombination and excision of the intervening sequence (Parks et al., 2004; Ryder et al., 2007). Following recombination and precise excision of the remaining hybrid transposon, the entire open reading frame of the *pldn* locus was removed (Fig. 1*A*; this allele referred to as *pldn^Δ1^*), and the genetic lesion was confirmed by PCR (Fig. 1*B*). *pldn^Δ1^* mutants were viable and fertile and could be maintained as healthy homozygous stocks.

Immunoblot analysis revealed that Pldn is expressed as a single band at 19 kDa (Fig. 1*C*), consistent with the predicted molecular weight of the lone isoform in the *Drosophila* genome. This 19-kDa band was absent in heads of *pldn^Δ1^* mutants, confirming the specificity of the Pldn antibody, and overexpression of a *UAS-pldn-3xflag* transgene revealed increased Pldn protein running at a slightly larger size, as expected with the 3xflag tag (Fig. 1*C*). Pldn was expressed in all stages examined (embryos through adults) and was present in larval and adult brain and body extracts (data not shown), consistent with *Drosophila* Pldn, like the vertebrate homolog, being broadly expressed. Together, this demonstrates that *pldn* is broadly expressed and that *pldn^Δ1^* is a null mutation.

### Pallidin localizes to presynaptic microtubules and is not required for synaptic growth or structure

In vertebrates, Pldn is associated with the AP-3 complex, F-actin, and Syntaxin 13 (Huang et al., 1999; Parast and Otey, 2000; Bang et al., 2001; Falcón-Pérez et al., 2002; Di Pietro et al., 2006; Delevoye et al., 2016), suggesting that Pldn may interact with both the cytoskeleton and endosomal structures, but whether this association holds at synapses is not known. We therefore examined the synaptic expression and localization of Pldn at the *Drosophila* NMJ. Pldn immunostaining revealed a specific signal in both presynaptic terminals of motor neurons as well as in postsynaptic muscles. In presynaptic terminals, Pldn immunostaining appeared to label neuronal microtubule structures, significantly overlapping with Futsch, a marker for neuronal microtubules (Hummel et al., 2000) (Fig. 1*D–F*). This signal was absent in *pldn^Δ1^* mutant synapses (Fig. 1*D*), confirming the specificity of this antibody and that *pldn^Δ1^* is a null mutation. In the postsynaptic muscle, Pldn appeared to localize to Z-disc structures (Fig. 1*G*), consistent with observations of Pldn association in vertebrate striated muscle (Bang et al., 2001; Parast and Otey, 2000). These Z-discs are cytoskeletal anchors for muscle filaments as well as signal transduction centers (Clark et al., 2002; Collin et al., 2012; Granger et al., 2014; Delevoye et al., 2016). We also noted that the normally organized F-actin bundles in the muscle, labeled with phalloidin, appeared disorganized in *pldn* mutants (Fig. 1*G*). Thus, Pldn is present in both pre- and postsynaptic compartments at the *Drosophila* NMJ, where it is associated with cytoskeletal structures.

**Figure 2.**
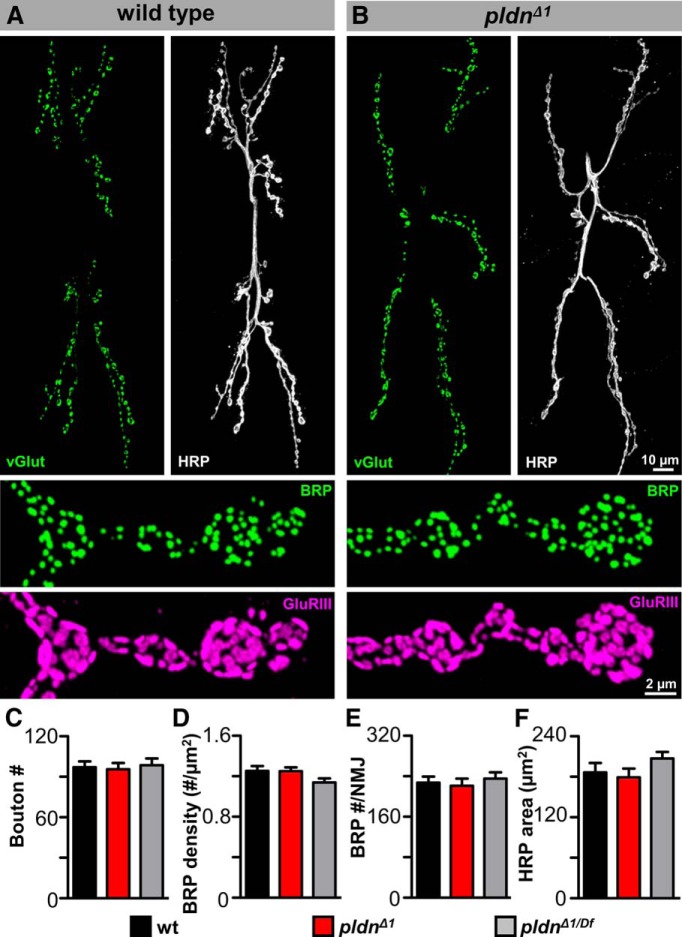
Synaptic growth and structure is unperturbed in *pallidin* mutants. Representative images of muscle 6/7 NMJs from wild type (*w^1118^*) (***A***) and *pldn^Δ1^* mutants (***B***) immunostained with anti-vGlut (synaptic vesicle marker; green), anti-HRP (white). Below, wild-type and *pldn^Δ1^* NMJs on muscle 4 immunostained with anti-BRP (active zone marker; green) and GluRIII (postsynaptic glutamate receptor marker; magenta). No significant differences are observed in bouton number (***C***), BRP density (***D***), BRP number/NMJ (***E***), or HRP area (***F***) in wild-type (*n* = 12), *pldn^Δ1^* (*n* = 12), and *pldn^Δ1/Df^* (*w^1118^*;*pldn^Δ1^*/*Df(3L)BSC675*; *n* = 10). *p* > 0.05; one-way ANOVA for all parameters.

Recent studies have suggested that *pldn* and other components of the BLOC-1 complex have roles in synaptic development (Ghiani et al., 2010; Wu et al., 2011; Zhou et al., 2012; Mullin et al., 2015). We therefore examined synaptic growth and structure in *pldn^Δ1^* mutants as well as in *pldn^Δ1^ in trans* with a deficiency (*pldn^Δ1^* ^/^*^Df^*). We immunolabeled the NMJ with antibodies specific to the presynaptic neuronal membrane (HRP), the postsynaptic density (discs large; DLG), presynaptic active zones (Bruchpilot; BRP), synaptic vesicles (Synapsin; SYN; vesicular glutamate transporter; vGlut), and postsynaptic glutamate receptors (GluRIII and GluRIIA). No major differences in synapse morphology or structure were observed in *pldn^Δ1^* mutants or *pldn^Δ1^* ^/^*^Df^*, nor did we note any changes in axon guidance or targeting of motor neurons to their proper target muscle (Fig. 2*A,B* and data not shown). To measure synaptic growth and structure, we quantified membrane surface area (HRP area), the number of synaptic boutons, and the density and numbers of active zones (Fig. 2*C–F*). We found no significant difference in any of these values in *pldn^Δ1^* mutants compared with wild type, nor did we observe any changes in the organization of postsynaptic glutamate receptors (Fig. 2*A*,*B* and data not shown). Thus, we conclude that *pldn* is not required for proper synaptic morphogenesis, growth, or architecture.

### Pallidin stability depends on the BLOC-1 components Dysbindin and Blos1

**Figure 3.**
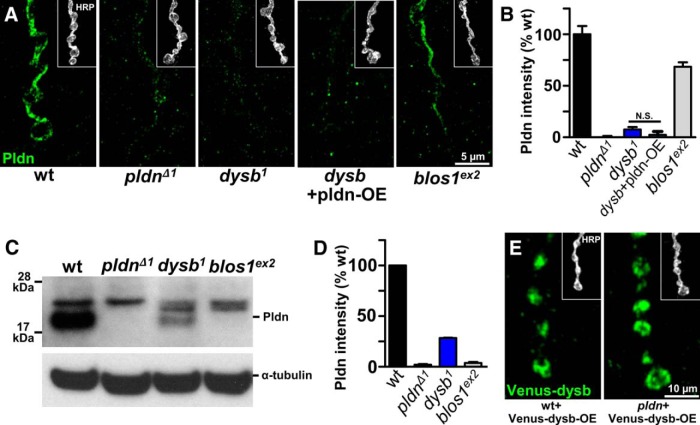
Pallidin stability is dependent on *dysbindin* and *blos1*. ***A***, Representative images of Pldn immunostaining (green) in NMJs of wild type, *pldn^Δ1^*, *dysb^1^*(*w^1118^*;*dysb^1^*), *dysb*+pldn-OE (*w^1118^*;*OK6*/*UAS-pldn-3xflag*;*dysb^1^*), and *blos1^ex2^* (*w^1118^*;*blos1^ex2^*) mutants. Inset, neuronal membrane (HRP; white). ***B***, Quantification of Pldn signal intensity, normalized to HRP intensity, for the indicated genotypes (*n* = 13-20). ***C***, Immunoblot analysis of adult head lysates probed for Pldn in wild type, *pldn^Δ1^*, *dysb^1^*, *blos1^ex2^*. ***D***, Quantification of Pldn immunoblot intensity normalized to α-tubulin for the genotypes indicated (*n* = 3). ***E***, Representative images of Venus-Dysb immunostaining (green) in NMJs of wt+venus-dysb-OE (*w^1118^*;*OK6*/+;*UAS-venus-dysb*/+) and *pldn*+venus-dysb-OE (*w^1118^*;*OK6*/+;*UAS-venus-dysb, pldn^Δ1/^pldn^Δ1)^*. The intensity of Venus-Dysb in *pldn*+venus-dysb-OE (1.74 ± 0.11 a.u., *n* = 15) is significantly increased compared with that of wt+venus-dysb-OE (0.91 ± 0.09 a.u., *n* = 9); *p* < 0.001; Student’s *t* test.

Biochemical studies of the BLOC-1 complex have demonstrated that the stability of some BLOC-1 components depend on the presence of other components, and Pldn protein levels are reduced in *dysbindin*, *cappuccino*, *muted*, and *blos3* mutants (Falcón-Pérez et al., 2002; Ciciotte et al., 2003; Li et al., 2003; Starcevic and Dell'Angelica, 2004). We therefore examined Pldn expression in the two other genetic mutations in BLOC-1 subunits that exist in *Drosophila*, *dysbindin* (*dysb*) (Dickman and Davis, 2009) and *blos1* (Cheli et al., 2010). We observed a reduction in Pldn immunolabeling at NMJ synapses in both mutants, with an almost complete loss of Pldn in *dysb^1^* mutants (92.4% reduction), and a more moderate reduction in *blos1^ex2^* mutants (31.5% reduction; Fig. 3*A,B*). Further, we examined Pldn protein stability by immunoblot of lysates from *dysb^1^* and *blos1^ex2^* mutant heads. Similarly, we observed a drastic loss of Pldn in *dysb^1^* mutants (71.6% reduction), with an even larger reduction in *blos1^ex2^* mutants (96.2% reduction; Fig. 3*C,D*). Given the large reduction of Pldn in *dysb* mutants, we asked whether overexpression of *pldn* in *dysb* mutants could overcome the dependency of Pldn stability on endogenous Dysb. Surprisingly, we observed no significant difference in Pldn immunostaining when *pldn* was neuronally overexpressed in *dysb* mutants (Fig. 3*A,B*). Finally, we asked whether the dependence of Pldn stability on *dysb* was reciprocal. To address this question, we overexpressed a *UAS-Venus-Dysbindin* transgene in neurons in a wild-type and *pldn* mutant background and immunostained NMJs for Venus-Dysbindin. We found no reduction in Venus-Dysbindin levels in *pldn* mutants compared with wild type (Fig. 3*E*). In fact, we observed a significant increase in Venus-Dysbindin expression in *pldn* mutants (1.91-fold increase; *p* < 0.001; Student’s *t* test), suggesting that Pldn may actually limit the stability of Dysbindin. Thus, Dysbindin and Pallidin protein stability are not reciprocally dependent on each other.

Together, these data demonstrate three important points about the dependency of Pldn stability on other BLOC-1 subunits. First, although Pldn stability is dependent on other BLOC-1 components, as observed biochemically (Falcón-Pérez et al., 2002; Ciciotte et al., 2003; Li et al., 2003; Gwynn et al., 2004), this dependence is not uniform, with Pldn at synaptic terminals appearing to be more sensitive to levels of *dysb* than to *blos1*. Second, Pldn levels at synaptic terminals, as determined by immunostaining, did not quantitatively correspond to the reduction in levels observed in whole head lysates by immunoblot, suggesting there may be differential dependency for Pldn stability in neuronal compartments and/or cell types. Third, at least in the case of Dysb stability, *pldn* does not exert the same control of stability on Dysb compared with the dependency of Pldn on *dysb*. Rather, Dysb levels are not reduced when overexpressed in *pldn* mutants, and appear to actually increase at synaptic terminals.

### Pallidin is dispensable for baseline neurotransmission and presynaptic homeostatic potentiation

**Figure 4.**
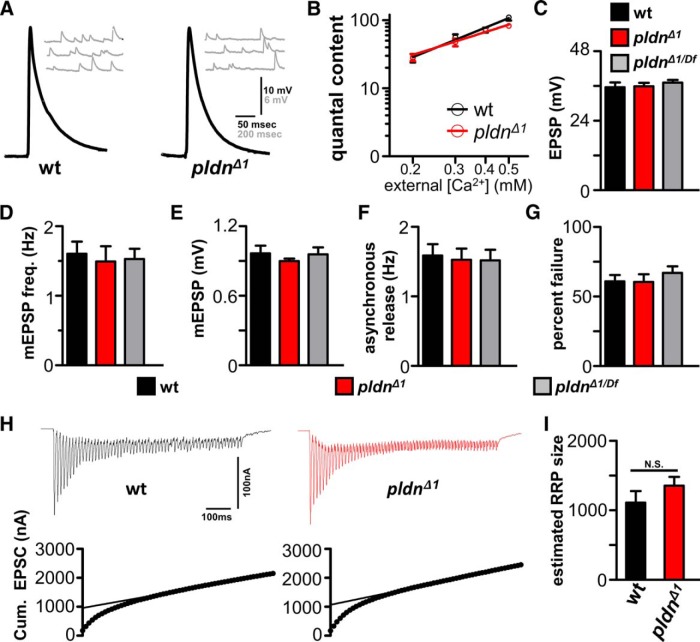
Baseline synaptic transmission is normal in *pallidin* mutants. ***A***, Representative electrophysiological traces (EPSP and mEPSP traces) from wild-type and *pldn^Δ1^* mutant synapses. ***B***, Quantal content was determined across a range of extracellular calcium concentrations for wild-type and *pldn^Δ1^* synapses. No significant difference in the slope of the line, indicating the apparent calcium cooperativity of synaptic transmission, was observed. No significant differences were observed in the EPSP amplitude (***C***), mEPSP frequency (***D***), mEPSP amplitude (***E***), asynchronous release (assayed by determining the mEPSP frequency within the 2 s immediately after EPSP stimulation) (***F***), or probability of release measured by failure analysis (assayed by % EPSP failure in 0.1mM Ca^2+^) (***G***) in wild-type (*n* = 10), *pldn^Δ1^* (*n* = 10), and *pldn^Δ1/Df^* (*n* = 10) mutant synapses. ***H***, Representative EPSC traces (top) and cumulative EPSC amplitudes (bottom) using two electrode voltage clamp evoked by 60-Hz stimulation (60 stimuli) in wild-type and *pldn^Δ1^*mutant synapses. No significant differences were observed in the estimated readily releasable synaptic vesicle pool (RRP) between wild type (*n* = 7) and *pldn^Δ1^* (*n* = 9) (***I***). *p* > 0.05; one-way ANOVA for all parameters.

Synaptic physiology has not been examined in *pallidin* mutants in any system, although changes in basal synaptic transmission have been observed in other BLOC-1 mutants (Numakawa et al., 2004; Chen et al., 2008; Dickman and Davis, 2009; Pan et al., 2009; Tang et al., 2009; Cheli et al., 2010). We therefore characterized synaptic physiology at the NMJ in *pldn^Δ1^* mutants and *pldn^Δ1^* ^/^*^Df^*, comparing values of miniature EPSP (mEPSP) frequency, mEPSP amplitude, evoked EPSP amplitude, and quantal content across a range of extracellular calcium conditions. We observed no major differences in mEPSP frequency, amplitude, or EPSP amplitudes in standard saline (0.4 mM extracellular Ca^2+^) in *pldn* mutants compared with controls (Fig. 4*A,C–E*). In addition, there was no change in the apparent calcium cooperativity of synaptic transmission, with *pldn^Δ1^* mutants releasing similar quantal content across a range of extracellular calcium concentrations compared with wild type (Fig. 4*B*). We went on to test the state of presynaptic function in further detail, examining asynchronous release, presynaptic release probability (failure analysis), and the size of the readily releasable synaptic vesicle pool. We observed no significant difference in asynchronous release (Fig. 4*F*), failure analysis (Fig. 4*G*), synaptic transmission at elevated extracellular calcium using two electrode voltage clamp (2 mM; Fig. 4*H*), or the estimated size of the readily releasable vesicle pool (Fig. 4*I*). Thus, surprisingly, *pldn* null mutants have no major defects in synaptic growth, structure, or baseline function, whereas changes in these processes have been reported in mutations in other BLOC-1 subunits.

The *Drosophila* NMJ has been established as a powerful model synapse to characterize presynaptic homeostatic plasticity (Frank et al., 2013; Davis and Müller, 2015). Using an acute pharmacological assay in which subblocking concentrations of the postsynaptic glutamate receptor antagonist philanthotoxin (PhTx) is applied to the dissected larval NMJ, mEPSP amplitude is reduced due to the irreversible binding of the toxin (Frank et al., 2006). However, EPSP amplitude is restored to baseline levels due to a rapid, homeostatic increase in presynaptic release (quantal content). The BLOC-1 components *dysbindin* and *snapin* have previously been shown to be required for this homeostatic increase in presynaptic release, where quantal content remains unchanged in these mutants after application of PhTx, leading to a reduced EPSP amplitude (Dickman and Davis, 2009). Given that PHP is blocked in two BLOC-1 components, we sought to determine whether PHP could be expressed over acute and chronic time scales in *pldn* mutants.

**Figure 5.**
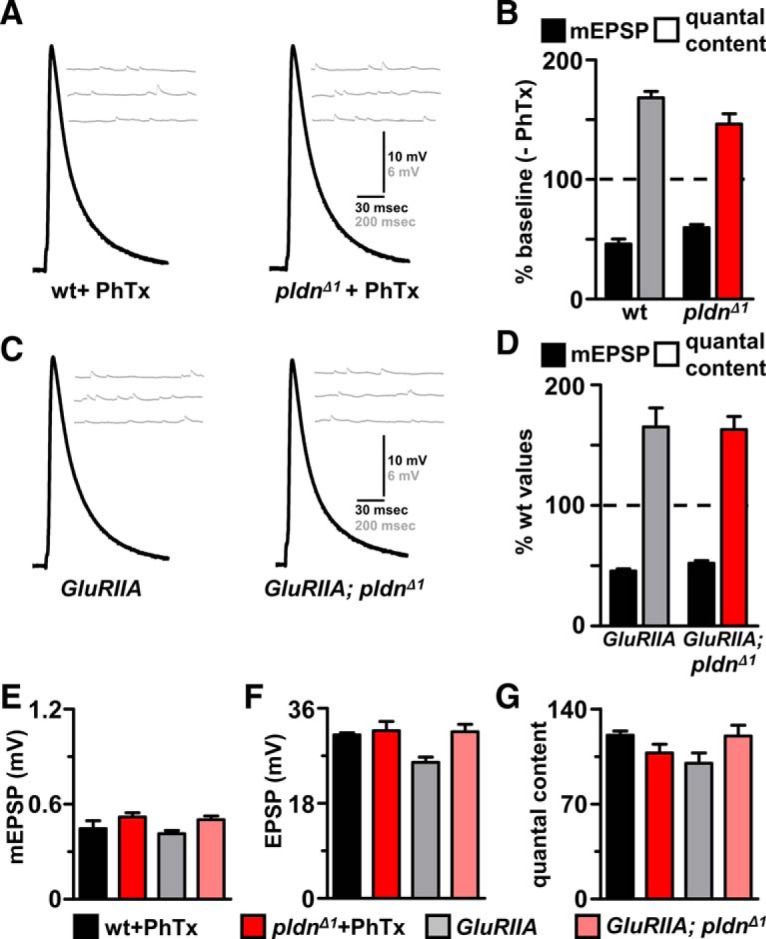
*pallidin* mutants retain the capacity to express presynaptic homeostatic potentiation. ***A***, Representative EPSP and mEPSP traces from wild-type and *pldn^Δ1^* mutant synapses after PhTx application (20 µM). ***B***, Normalized mEPSP amplitude and quantal content values of wild-type (*n* = 10) and *pldn^Δ1^* (*n* = 12) mutants following PhTx application. Data are normalized to values of each genotype in the absence of PhTx. No deficit in acute presynaptic homeostatic potentiation was observed in *pldn^Δ1^* mutants. ***C***, Representative EPSP and mEPSP traces from *GluRIIA* (*w^1118^;GluRIIA^sp16^*) and *GluRIIA*;*pldn^Δ1^* (*w^1118^*;*GluRIIA^sp16^*;*pldn^Δ1^*) mutant synapses. ***D***, Normalized mEPSP amplitude and quantal content values of *GluRIIA* (*n* = 18) and *GluRIIA*;*pldn^Δ1^* (*n* = 8). Data are normalized to wild-type values. No deficit in chronic presynaptic homeostatic potentiation was observed in *pldn^Δ1^* mutants. ***E***, Absolute values of mEPSP amplitude, EPSP amplitude (***F***), and quantal content (***G***) from the normalized data in *B* and *D* for the indicated genotypes.

We applied PhTx to *pldn^Δ1^* NMJs and measured mEPSP amplitude, EPSP amplitude, and calculated quantal content. Although mEPSP amplitudes were reduced to similar levels in wild-type and *pldn* mutants due to the acute blockade of postsynaptic glutamate receptors by PhTx (Fig. 5*E*), EPSP amplitudes were maintained and PHP was robustly expressed (Fig. 5*A,B,F*). We then tested whether synaptic homeostasis was expressed normally in *pldn^Δ1^* mutations when chronically induced due to genetic loss of the postsynaptic glutamate receptor *GluRIIA* throughout development, which normally triggers a homeostatic increase in presynaptic release (Petersen et al., 1997). Similar to the acute induction and expression of PHP, PHP is robustly expressed in *GluRIIA*;*pldn* mutants (Fig. 5*C,D*). Thus, *pldn* is dispensable for both the acute induction and chronic expression of PHP.

### *pallidin* is necessary to maintain and recover the synaptic pool during high activity

Although we did not observe any major changes in synaptic function under basal conditions in *pldn* mutants, we asked whether an important function of *pldn* may be revealed under conditions of synaptic stress. BLOC-1 components have been implicated in endosomal sorting in a variety of tissues, and we considered that during high levels of activity, the importance of *pldn* at synapses may be revealed. During these conditions, membrane trafficking at synapses must be rapidly and accurately orchestrated to ensure proper endocytosis, sorting, regeneration, and mobilization of synaptic vesicles to maintain the functional vesicle pool and sustain neurotransmission. Indeed, the importance of trafficking and sorting at synaptic endosomes would be highlighted in this condition, and we reasoned that by stressing the synapse through high-intensity stimulation, we may reveal a role for *pldn* that would not be apparent at rest.

**Figure 6.**
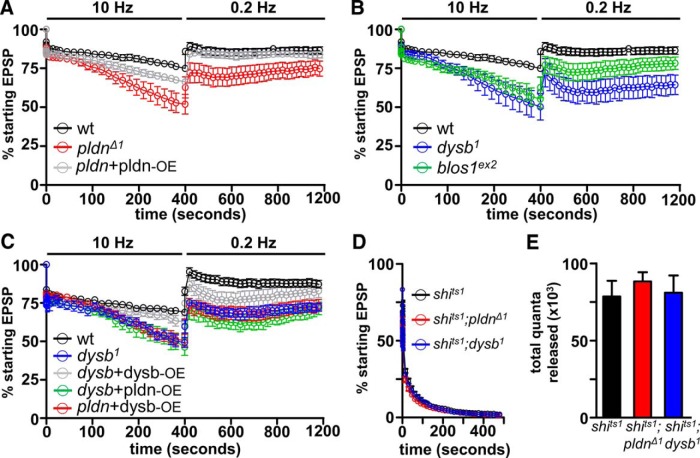
BLOC-1 mutants fail to sustain neurotransmitter release during high-frequency stimulation. ***A***, Increased rate of depletion and slowed recovery of the synaptic vesicle pool is observed under high-frequency stimulation in *pldn^Δ1^* mutants (*n* = 15; *p* < 0.01; Student’s *t* test) compared with wild type (*n* = 11). Presynaptic overexpression of *pallidin* in *pldn^Δ1^* mutants (*pldn*+pldn-OE: *w^1118^;OK6-Gal4/UAS-pldn;pldn^Δ1^*; *n* = 12) significantly slows the rate of depletion and increases the rate of recovery. Synapses were stimulated at 10 Hz in 2 mM extracellular calcium for 10 min, then allowed to recover, taking a test pulse at 0.2 Hz for the following 10 min. EPSP amplitudes for each time point were binned for 2 s, normalized to prestimulus amplitudes, and plotted as a function of time. ***B***, A similar increase in depletion and slowing of recovery is observed in *dysb^1^* (*n* = 9; *p* < 0.05; Student’s *t* test) and *blos1^ex2^* mutants (*n* = 13; *p* < 0.05; Student’s *t* test). ***C***, Both overexpression of *dysb* in *pldn^Δ1^* mutants (*pldn*+dysb-OE: *w^1118^*;*OK6-Gal4*/*UAS-3xflag-dysb*;*pldn^Δ1^*, *n* = 12) and overexpression of *pldn* in *dysb^1^* mutants (*dysb*+pldn-OE: *w^1118^*;*OK6-Gal4*/*UAS-pldn-3xflag*;*dysb^1^*, *n* = 12) fail to rescue the increased rundown during high-frequency stimulation. ***D***, Determination of the total releasable synaptic vesicle pool. Control (*shi^ts1^*, *n* = 6), *pldn^Δ1^* (*shi^ts1^*;*pldn^Δ1^*, *n* = 5), and *dysb^1^* (*shi^ts1^*;*dysb^1^*, *n* = 5) mutants were stimulated at 10 Hz in 2 mM calcium at 32°C to deplete the total releasable synaptic vesicle pool. EPSP amplitudes at each time point were plotted as a percentage of the starting EPSP amplitude. ***E***, No significant difference was observed in the total quanta released between the three genotypes. *p* > 0.05; one-way ANOVA.

Under conditions of high extracellular calcium, we first stimulated NMJs at 10 Hz for 10 min to deplete the synaptic vesicle pool, then measured recovery of the pool for an additional 10 min, taking a test pulse every 5 s. Following an initial rapid depletion typically observed in the initial seconds of stimulation, wild-type synapses largely maintained synaptic vesicle release for the duration of the stimulus, ending at ∼80% of the starting EPSP amplitude and rapidly recovering to above 90% of prestimulus amplitudes (Fig. 6*A*). In contrast, this stimulation protocol revealed a more rapid rundown of the synaptic vesicle pool in *pldn* mutants, ending at ∼45% of the starting EPSP amplitude (Fig. 6*A*). Further, *pldn* mutants failed to fully replenish the depleted synaptic vesicle pool over the course of the next 10 min of recovery following high-frequency stimulation, only recovering to ∼60% of prestimulus amplitudes (Fig. 6*A*). This level of depletion was significantly reduced when a *pldn* transgene was expressed in motorneurons in a *pldn* mutant background, demonstrating that *pldn* is required presynaptically to maintain the rapidly recycling synaptic vesicle pool.

Given the reduction in Pldn protein levels in both *dysb^1^* and *blos1^ex2^* mutants, we considered whether these mutants share a similar deficit in maintaining the rapidly recycling synaptic vesicle pool. Using the same stimulation paradigm, we observed a similar rundown in both *dysb^1^* and *blos1^ex2^* mutants, as well as a delayed recovery of the depleted synaptic vesicle pool (Fig. 6*B*). Thus, *pldn is* necessary for both the sustainment and recovery of the synaptic vesicle pool during and following high levels of activity, a phenotype shared in *dysb^1^* and *blos1^ex2^* mutants, which also exhibits a marked reduction in Pldn expression.

Next, we sought to determine whether the deficit in maintaining the functional vesicle pool during high activity in *dysb* mutants was due to loss of Pldn itself, or rather whether Dysb may have a direct role in maintaining the recycling vesicle pool. To distinguish between these possibilities, we neuronally overexpressed *pldn* in *dysb* mutants and overexpressed *dysb* in *pldn* mutants. As anticipated by the failure to restore Pldn protein levels at synapses in *dysb* mutants (Fig. 3*A*,*B*), the rapid rundown of the vesicle pool failed to be rescued in either of these conditions (Fig. 6*C*). However, we were able to restore the recycling vesicle pool by overexpressing *dysb* in *dysb* mutants (Fig. 6*C*). As we have demonstrated that overexpressed Dysb levels were not reduced in *pldn* mutants (Fig. 3*E*), this indicates that loss of Pldn itself, and not other BLOC-1 components, likely explains the failure to maintain the recycling vesicle pool in *dysb* mutants.

Finally, we considered the possibility that *pldn* may not be necessary for synaptic vesicle recycling *per se*, but rather for establishing the full size of the starting synaptic vesicle pool. In principle, this pool might be reduced in *pldn* mutants, given the roles of *pldn* and BLOC-1 in vesicle biogenesis (Di Pietro et al., 2006; Delevoye et al., 2016). Indeed, a reduction in the starting vesicle pool could explain the more rapid rundown of the synaptic vesicle pool without necessitating any additional role for Pldn in synaptic vesicle recycling. To determine the size of the entire releasable synaptic vesicle pool, we took advantage of the temperature-sensitive mutation in the *Drosophila dynamin* gene, *shibire* (shi). Although synaptic transmission is normal at room temperature in these mutants, all synaptic vesicle endocytosis ceases at restrictive temperatures (32°C) due to a disruption in the ability of Dynamin to drive fission of synaptic vesicles and replenish the vesicle pool (Kuromi and Kidokoro, 1998; Delgado et al., 2000). We measured synaptic transmission at the restrictive temperature in *shi* mutants alone, as well as in *shi*;*pldn* and *shi*;*dysb* double mutants (Fig. 6*D*). In *shi* mutants alone, full depletion of the entire synaptic vesicle pool was achieved after ∼400 s of stimulation at 15 Hz in 2 mM extracellular calcium. Transmission ceases because every releasable vesicle is lost without the replenishment of new synaptic vesicles due to this complete block of endocytosis. Importantly, both *shi*;*pldn* and *shi*;*dysb* mutants also showed similar rates of depletion of the releasable synaptic vesicle pool (Fig. 6*D*). We calculated the total quanta released in each mutant until full depletion, finding that all genotypes had ∼75,000 total quanta, with no significant differences between the genotypes (Fig. 6*E*). Thus, *pldn* is not required for the biogenesis or establishment of a full initial synaptic vesicle pool size, but rather is necessary to rapidly replenish depleted synaptic vesicles during and following high levels of activity.

### High synaptic activity depletes FYVE-positive endosomes in *pallidin* mutants

A variety of dynamic endosomal structures are known to exist at the presynaptic terminal, where they are involved in modulating diverse aspects of synaptic growth signaling, membrane trafficking and exchange, and synaptic vesicle recycling (Wucherpfennig et al., 2003; Rodal and Littleton, 2008; Uytterhoeven et al., 2011; Saheki and De Camilli, 2012; Kononenko and Haucke, 2015; Deshpande and Rodal, 2016). Given the inability of *pldn* mutants to sustain the synaptic vesicle pool during high-frequency stimulation, and the associations of BLOC-1 in controlling endosomal sorting and trafficking, we considered whether endosomal dysfunction may contribute to the failure to sustain the vesicle pool in *pldn* mutants. In particular, we focused on endosomal structures known to participate in synaptic vesicle recycling. One key endosome at the synapse that has been characterized in significant detail at the *Drosophila* NMJ are Rab5-positive early endosomes. These distinct structures are defined by specific labeling with the small GTPase Rab5 (Wucherpfennig et al., 2003; Rodal et al., 2011), and are enriched in the phospholipid phosphatidylinositol-3-phosphate (PI[3]P). PI[3]P specifically binds to the FYVE zinc-finger domain of endosomal factors such as the Rab5 effectors EEA1 and Rabeenosyn-5 (Stenmark et al., 1995;; Lawe et al., 2000; Nielsen et al., 2000; Wucherpfennig et al., 2003). These endosomes serve as sorting stations for synaptic vesicles, directing proteins and membrane to distinct intracellular compartments, including pathways for degradation or the genesis of new synaptic vesicle pools (Wucherpfennig et al., 2003; Uytterhoeven et al., 2011). Ultimately, these key endosomal sorting stations help to maintain the synaptic vesicle pool during high activity, even disappearing when endocytosis from the plasma membrane is blocked due to acute inactivation of *shibire* (Wucherpfennig et al., 2003).

**Figure 7.**
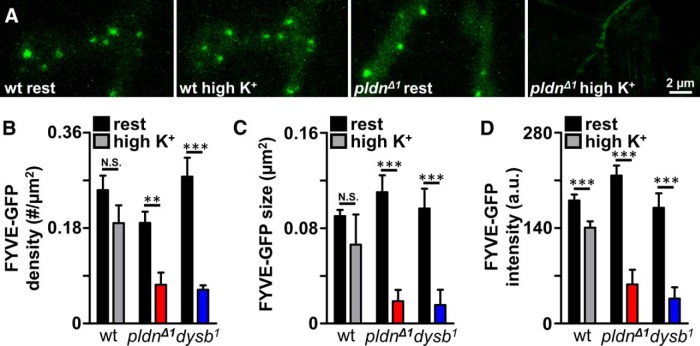
Activity-dependent loss of FYVE-positive synaptic endosomes in *pallidin* mutants. ***A***, GFP-2xFYVE puncta are observed in synapses from control (*w^1118^;OK6-Gal4/UAS-GFP-myc-2xFYVE*) and *pldn^Δ1^* mutants (*w^1118^;OK6-Gal4/UAS-GFP-myc-2xFYVE*; *pldn^Δ1^*) at rest and following a 5-min incubation in 90 mM KCl (high K^+^). Similar GFP-2xFYVE density and intensity are observed in controls before and after stimulation, whereas *pldn^Δ1^* NMJs have reduced GFP-2xFYVE density and intensity following stimulation. Quantification of GFP-2xFYVE density (***B***), size (***C***), and intensity (***D***) in wild-type (*n* = 13), *pldn^Δ1^* (*n* = 8), and *dysb^1^* mutants (*w^1118^;OK6-Gal4/UAS-GFP-myc-2xFYVE*; *dysb^1^*; *n* = 9) at rest and following high K^+^ stimulation. *p < 0.05; ** *p* < 0.01; ***p < 0.001; paired Student’s *t* test.

To determine the dynamics and functionality of Rab5-positive endosomal structures in the absence of Pldn, we characterized the number and maintenance of these endosomes. First, we expressed GFP-2xFYVE in motor neurons and examined the punctate endosomal structures in synaptic boutons at the NMJ that has been observed by others (Wucherpfennig et al., 2003; Rodal et al., 2011). At rest, FYVE-positive endosomes in *pldn* mutants showed similar size and density compared with wild type, although *pldn* mutants displayed a slight reduction in the density of these structures (Fig. 7*A,B*). We then subjected the NMJ to stimulation with 90 mM KCl for 5 min and found that FYVE-positive endosomes in wild-type terminals maintained their integrity, showing no significant changes in density or size, and a small but significant reduction in fluorescence intensity (Fig. 7). In contrast, GFP-2xFYVE labeled endosomes were greatly reduced following high K^+^ stimulation in *pldn* mutants, exhibiting large reductions in the density, size, and intensity of GFP-2xFYVE puncta (Fig. 7), with a significant fraction disappearing altogether. Similar results were observed in *dysb* mutants, consistent with the loss of Pldn and reduced capacity to maintain the recycling vesicle pool in these mutants (Fig. 7*B–D*). Together, these experiments demonstrate that the activity-dependent maintenance of FYVE-positive early endosomal structures depends on Pldn.

### Tubular endosomal structures emerge in *pallidin* mutant synapses following high activity

**Figure 8.**
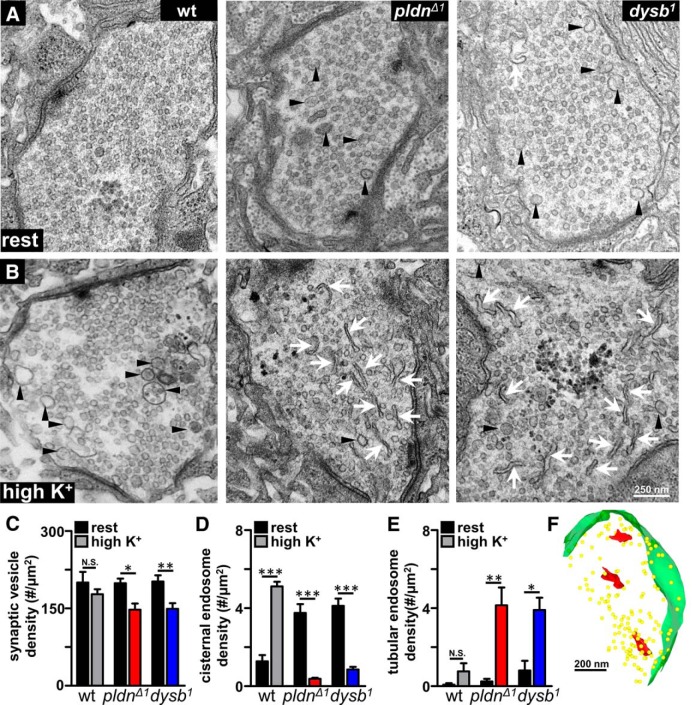
Activity-dependent accumulation of tubular endosomal structures in *pallidin* mutants. ***A***, Representative electron micrographs of NMJs at rest in wild-type, *pldn^Δ1^*, and *dysb^1^*mutants. ***B***, Increased tubular endosomal structures are observed in BLOC-1 mutants following incubation in high K^+^ (90 mM K^+^, 5 min), while rarely observed in controls. Tubular endosomes (white arrows) and cisternal endosomes (black arrows) are noted. Quantification of the density of synaptic vesicles (***C***), cisternal endosomes (***D***), and tubular endosomes (***E***) at rest and following high K^+^ stimulation in wild type (*n* = 16 rest and high K^+^), *pldn^Δ1^* (*n* = 13 rest and *n* = 28 high K^+^), and *dysb^1^* (*n* = 7 rest and *n* = 28 high K^+^). Note that synaptic vesicle and cisternal endosome densities are reduced, while the tubular endosome density is increased in *pldn^Δ1^* and *dysb^1^* mutants following stimulation. ***F***, Three-dimensional serial EM reconstruction of tubular endosomal structures (red) near synaptic vesicles (yellow), demonstrating they are not continuous with the plasma membrane (green). *p < 0.05; **p < 0.01; ***p < 0.001; Student’s *t* test.

Given the inability to sustain neurotransmission during high-frequency stimulation in *pldn* mutants, as well as the deficits in maintaining FYVE-positive endosomal structures following activity, we considered whether visualization of synaptic ultrastructure may reveal details about the state of endosomal structures during activity that could not be discerned through confocal imaging alone. At rest, overall synaptic ultrastructure appeared relatively consistent between wild-type, *pldn*, and *dysb* genotypes, each showing similar levels of active zones and T bars, and no significant differences in the size, number, and density of synaptic vesicles at NMJ boutons (Fig. 8*A* and data not shown). However, cisternal endosomal structures, defined as clear vesicles >80 nm in diameter, were increased in both *pldn*, and *dysb* mutants at rest (Fig. 8*A,C*), suggesting an accumulation of newly formed vesicular structures (Heuser and Reese, 1973; Körber et al., 2012). These are typically transient structures, and cisternal endosomes were observed to accumulate in wild-type synapses following activity (Fig. 8*B,D*). Interestingly, a similar accumulation of cisternal endosomes at rest were reported in *Rab5* mutants (Wucherpfennig et al., 2003), consistent with defects in synaptic vesicle endocytosis and, perhaps, Rab5-dependent endosomal trafficking of recycling synaptic vesicles.

To determine how endosomal structures are altered following high levels of activity and synaptic vesicle recycling, we subjected control, *pldn*, and *dysb* genotypes to depolarization in 90 mM KCl for 5 min, followed by immediate fixation and preparation for electron microscopy (see Materials and Methods). Following stimulation, wild-type NMJs exhibited no significant change in synaptic vesicle density and an increase in cisternal endosomal structures (Fig. 8*B–D*), consistent with increased rates of endocytosis, as observed in other studies (Akbergenova and Bykhovskaia, 2009). In contrast, analysis of NMJs in both *pldn*, and *dysb* mutants revealed a significant decrease in synaptic vesicle density and cisternal endosomal structures, consistent with reduced recycling rates (Fig. 8*B–D*). However, the most striking change observed in *pldn* and *dysb* synapses following activity was the emergence of tubular endosomal structures (Fig. 8*B,E*). These structures are rarely observed in wild type, but have been observed at the *Drosophila* NMJ when Rab5 activity is perturbed (Wucherpfennig et al., 2003). Considering that elevated activity leads to a reduction in FYVE-positive endosomes in *pldn* and *dysb* mutants, these tubular structures may be related to a diminishment of PI[3]P-enriched synaptic endosomes and a concomitant expansion of the endosomal system.

Finally, we considered the possibility that these tubular endosomal structures were not actually intracellular endosomes but rather were invaginations from the plasma membrane. These invaginations are indicative of an "activity-dependent bulk endocytosis" pathway that can be triggered when other forms of endocytosis are disrupted (Heerssen et al., 2008; Kasprowicz et al., 2008; Verstreken et al., 2009; Wu et al., 2014), and have also been observed in *dynamin* mutants (Wu et al., 2014). To test whether the tubular endosomes we observed were continuous with the plasma membrane, we performed two experiments. First, a bulk endocytosis assay utilizes dextran uptake to reveal synaptic uptake through the activity-dependent bulk endocytic pathway (Clayton and Cousin, 2009b; Uytterhoeven et al., 2011). We performed this assay but did not observe any change in bulk endocytosis levels between wild-type, *pldn*, and *dysb* mutants (data not shown). In addition, we performed a 3D serial reconstruction using electron microscopy of the tubular endosomal structures that emerged following high activity in *pldn* mutants. This reconstruction revealed that these structures are entirely cytosolic and discontinuous with the plasma membrane (Fig. 8*F*). Together, these results demonstrate that Pldn is necessary during conditions of high synaptic activity to rapidly transition membrane trafficking of synaptic vesicles through key endosomal intermediaries. In the absence of Pldn, tubular endosomal compartments emerge with a concomitant reduction in FYVE-positive early endosomes, leading to a decrease in the rapid and efficient recycling and recovery of the synaptic vesicle pool.

## Discussion

We have generated null mutations in *pallidin*, a central component of BLOC-1, and characterized synaptic structure and physiology in these mutants. Pldn is present at presynaptic terminals, where it localizes to synaptic microtubules and the cytoskeleton. We find that while *pallidin* does not have major roles in synaptic growth, structure, or function under basal conditions, *pallidin* is crucial to maintain the releasable synaptic vesicle pool during conditions of high activity. During these conditions, tubular endosomal structures accumulate with loss of Pldn, while FYVE-positive endosomes are reduced. We also find that the stability of Pldn depends crucially on the BLOC-1 subunits *dysbindin* and *blos1* and that mutations in these subunits phenocopy *pallidin* mutants, as expected due to destabilization of the protein. Together, our data demonstrate that although *pallidin* has no obvious roles in basal synaptic development and function, *pallidin* has a critical role during adaptive responses to synaptic activity by promoting the efficient trafficking and re-formation of synaptic vesicles through FYVE-positive endosomes.

### Synaptic functions of Pallidin at the *Drosophila* NMJ

We find no major alterations in synaptic development or transmission in *pallidin* mutants. Further, *pallidin* mutants are viable and healthy, and although we cannot rule out more subtle phenotypes or differences between species or systems, it is surprising how unperturbed synapses are in *pallidin* null mutants during basal conditions. In contrast, previous studies have reported moderate changes in synaptic growth, baseline function, and homeostatic plasticity in genetic mutations of other BLOC-1 subunits (Dickman and Davis, 2009; Ghiani et al., 2010; Shao et al., 2011; Wu et al., 2011; Dickman et al., 2012; Zhou et al., 2012; Mullin et al., 2015). For example, baseline synaptic transmission at lowered extracellular calcium is reduced in *dysbindin* mutants (Dickman and Davis, 2009), yet no such effect is observed in *pldn* mutants (Fig. 4). In addition, although both *dysbindin* and *snapin* are required for acute and chronic forms of synaptic homeostasis in *Drosophila* (Dickman and Davis, 2009; Dickman et al., 2012), no defects in presynaptic homeostatic plasticity were found in *pldn* mutants (Fig. 5). Similarly, *blos1* mutants were also found to robustly express homeostatic plasticity (Dickman et al., 2012), in contrast to *dysbindin* and *snapin* mutants. This suggests that despite being a central part of the BLOC-1 complex, genetic distinctions in synaptic function exist between *pallidin* and other components in *Drosophila*. This may be due to partial redundancy and complex gene dosage interactions between BLOC-1 components, as were recently reported (Larimore et al., 2014; Mullin et al., 2015).

Our data suggest that a core function of Pallidin at synapses is to promote the rapid and efficient maintenance of the functional synaptic vesicle pool under conditions of high activity. We found no evidence that *pldn* controls synaptic vesicle biogenesis, as may have been anticipated for the BLOC-1 complex, because we did not observe any change in the total releasable synaptic vesicle pool (Fig. 6). Instead, *pldn* is necessary for efficient synaptic vesicle trafficking during conditions of high activity, when at least a subset of synaptic vesicles are guided through endosomal intermediates for sorting, maintenance, and re-formation of critical functional constituents (Wucherpfennig et al., 2003; Jovic et al., 2010; Uytterhoeven et al., 2011; Fernandes et al., 2014). In addition, we observed a striking increase in tubular endosomal structures following activity. These tubular endosomes are rarely if ever observed in wild-type synapses and do not appear at rest in *pldn* mutants. Interestingly, similar structures have been observed when Rab5 activity is manipulated, when overexpression or dominant negative forms of Rab5 lead to the appearance of similar tubular endosomal structures (Shimizu et al., 2003; Wucherpfennig et al., 2003). These tubular structures are likely the result of an expanded and defective synaptic endosomal system due to abnormal regulation of Rab5 activity; such activities have been reported for other BLOC-1 components (John Peter et al., 2013; Rana et al., 2015). Indeed, there appears to be an intimate relationship between neuronal activity, Pldn, and Rab5 in synaptic vesicle trafficking, as loss of *pldn* leads to a reduction of FYVE/Rab5-positive endosomes following activity (Fig. 7). More generally, these findings point to a role for *pldn* and other BLOC-1 components having important and yet distinct functions at synapses during adaptive responses to neuronal stress, such as intense stimulation and homeostatic challenge to neurotransmission.

### Pallidin localization, stability, and the BLOC-1 complex

Pldn is ubiquitously expressed and localizes to cytoskeletal and endosomal structures (Huang et al., 1999; Parast and Otey, 2000; Bang et al., 2001; Falcón-Pérez et al., 2002; Di Pietro et al., 2006). At the *Drosophila* NMJ, Pldn localized to Z-bands in the postsynaptic muscle (Fig. 1*G*). These structures are a major cytoskeletal component of muscles that anchors actin filaments to enable muscle contraction (Clark et al., 2002; Collin et al., 2012; Granger et al., 2014; Delevoye et al., 2016). It is worthwhile to note that these muscle Z-bands appear disorganized in *pldn* mutants, suggesting that Pldn is required for the integrity or organization of these structures. In the presynaptic terminal, Pldn exhibits a high degree of colocalization with the neuronal microtubule marker Futsch (Fig. 1*D*). Given the role of *pldn* in promoting synaptic vesicle trafficking, this suggests that Pldn may coordinate interactions between synaptic vesicles and the cytoskeleton. Indeed, proper coordination between adaptors and the cytoskeleton is particularly important at synaptic terminals during synaptic vesicle recycling (Ferguson and De Camilli, 2012; Kononenko and Haucke, 2015; Delevoye et al., 2016). Interestingly, two other BLOC-1 subunits, Dysbindin and Snapin, colocalize with synaptic vesicle markers, not cytoskeletal structures (Dickman and Davis, 2009; Dickman et al., 2012). Given the disparate roles in transmission, homeostatic plasticity, and synaptic vesicle endocytosis observed between BLOC-1 subunits, it is tempting to speculate that these distinctions in localization are related to their different functions at synapses.

A variety of studies have noted the apparent unitary nature of the BLOC-1 complex, with significant biochemical evidence that the entire complex associates together as a single entity (Falcón-Pérez et al., 2002; Starcevic and Dell'Angelica, 2004; Setty et al., 2007; Larimore et al., 2014; Delevoye et al., 2016). Further, genetic reductions of some BLOC-1 subunits lead to the biochemical destabilization of other subunits (Falcón-Pérez et al., 2002; Zhang et al., 2002; Ciciotte et al., 2003; Li et al., 2003; Gwynn et al., 2004; Starcevic and Dell'Angelica, 2004; Setty et al., 2007; Feng et al., 2008; Ghiani and Dell'Angelica, 2011; Larimore et al., 2014; Delevoye et al., 2016). For example, Dysbindin and Muted are reduced by up to 60% and 90% in brain lysates from *pldn* mutants in mice (Li et al., 2003). However, complex interactions between BLOC-1 subunits have been reported (Larimore et al., 2014; Mullin et al., 2015), suggesting simple loss-of-function analysis may not always explain important functional properties of the BLOC-1 complex. Further, there is evidence for biochemical subcomplexes of the BLOC-1, in which Pallidin/Blos1/Cappucino and Dysbindin/Snapin/Blos2 exhibit differential association from the entire BLOC-1 complex (Lee et al., 2012). Consistent with these observations, we have found that protein levels of Pldn appear highly dependent on Dysb, where Pallidin is almost completely absent in *dysb* mutants, and even overexpression of *pldn* in *dysb* mutants fails to restore levels Pldn (Fig. 3*A*,*B*). However, this dependence is not reciprocal: *dysbindin* overexpression in *pldn* mutants led to no reduction in Dysb levels; if anything, an increase in Dysb was observed (Fig. 3*E*). Finally, it is loss of Pldn at synapses, and not a function for Dysb itself, which was necessary for rapid endocytosis, since overexpression of *dysb* in *pldn* mutants failed to rescue this phenotype (Fig. 6*C*).

Interestingly, although Pldn is also reduced in *blos1^ex2^* mutants, this reduction is not as severe as observed in *dysb* mutants at presynaptic NMJ terminals. Indeed, this distinction in stability may also be influenced by subcellular expression and/or trafficking of Pldn, as overall levels of Pldn, assessed through immunoblots of whole head lysates, were quantitatively different from levels assessed by immunostaining at the NMJ (Fig.3). Notably, there is some precedence for distinct genetic roles for BLOC-1 components in rodents. The changes in coat color observed in *pallidin*, *dysbindin*, and other BLOC-1 mutants are not exactly the same and have not been reported in *snapin* mutants (Tian et al., 2005), nor have the neurodegenerative defects reported in *snapin* mutants (Cai et al., 2010; Tian et al., 2005) been observed for other BLOC-1 mutants. Ultimately, BLOC-1 components at synapses appear to be involved in membrane trafficking, with complex interrelationships between individual components in regulating protein stability and functions in adaptive responses to synaptic stress.

## Synthesis

Reviewing Editor: Christian Lüscher, University of Geneva Decisions are customarily a result of the Reviewing Editor and the peer reviewers coming together and discussing their recommendations until a consensus is reached. When revisions are invited, a fact-based synthesis statement explaining their decision and outlining what is needed to prepare a revision will be listed below.

Two points should be addressed before publication of this interesting manuscript:

1) Is there a role of Pallidin during synaptic homeostasis?

It remains absolutely unclear why the authors did not test or comment on the potential role of Pallidin for synaptic homeostasis. The lab previously demonstrated that two other members of the Bloc-1 complex, Dysbindin (Dysb) and Snapin are critical for synaptic homeostasis (Dickman and Davis, 2009; Dickman et al., 2012). Similar to Pallidin mutations in dysb and snapin do not significantly alter baseline transmission or synapse development but are required for homeostatic stabilization of neuronal function. As Pallidin protein levels are dramatically reduced in dysb mutants this loss of Pallidin may be the critical component altered in dysb mutants. Both rapid synaptic homeostasis and genetically induced homeostasis should be analyzed.

2) Are Dysb or Snapin level altered in pallidin mutants?

The authors propose that the loss of Pallidin in dysb and blos1 mutants is causative for the observed defects in synaptic vesicle endocytosis in these mutants. However, as it has been previously demonstrated that Dysbindin levels are dramatically reduced in brains of pallidin mutant mice (Li et al., 2003), an equally valid hypothesis would be that a reduction of Dysb protein levels in pallidin mutants is responsible for the observed phenotypes. Ideally, Dysb and Blos1 protein level should be analyzed in pallidin mutants. As Dysb and Blos1 antibodies are likely not available (based on Dickman et al., 2009) alternative experiments should be considered. Is it possible to rescue the synaptic vesicle endocytosis defect of dysb mutants by overexpression of pallidin? If ectopic expression of pallidin can at least partially restore Pallidin protein levels and endocytosis it would provide substantial support for the presented model. Vice versa, dysb should be expressed in pallidin mutants (also tagged versions to address localization and level of Dysb). Extension of these experiments to synaptic homeostasis (see above) would be ideal.

Major points:

1. The authors show that basic neurotransmission is unaltered but deficiency is apparent during sustained stimulation. It would be important to know whether other forms of release, such as asynchronous release are affected. Do the authors have any data regarding potential changes i asynchronous release? They might already have this data from experiments where they used long stimuli at the end of the traces.

2. There are few other important parameters that would be important to know in order to get a full picture of the potential changes in vesicle dynamics. These are the probability of release, the size of the readily releasable pool.

Minor points:

1. How was quantal content calculated? Please provide relevant equations.

2. The flow of the manuscript would be better if the electrophysiology sections were followed each other (basic + sustained stem.).
